# Real-Time Particle Mass Spectrometry Based on Resonant Micro Strings

**DOI:** 10.3390/s100908092

**Published:** 2010-08-27

**Authors:** Silvan Schmid, Søren Dohn, Anja Boisen

**Affiliations:** Department of Micro- and Nanotechnology, Technical University of Denmark, DTU Nanotech, Building 345 East, DK-2800 Kongens Lyngby, Denmark; E-Mails: soren.dohn@nanotech.dtu.dk (S.D.); anja.boisen@nanotech.dtu.dk (A.B.)

**Keywords:** mass spectrometry, MEMS, NEMS, nanoparticles, string resonator, micro beads, aerosol, particle sensor

## Abstract

Micro- and nanomechanical resonators are widely being used as mass sensors due to their unprecedented mass sensitivity. We present a simple closed-form expression which allows a fast and quantitative calculation of the position and mass of individual particles placed on a micro or nano string by measuring the resonant frequency shifts of the first two bending modes. The method has been tested by detecting the mass spectrum of micro particles placed on a micro string. This method enables real-time mass spectrometry necessary for applications such as personal monitoring devices for the assessment of the exposure dose of airborne nanoparticles.

## 1. Introduction

In 1995 the use of resonant micrometer scaled cantilevers as mass sensors was proposed [1,2]. Since then, cantilever based mass sensors have been shown to have the sensitivity to measure the mass of single cells and large molecules [3–5]. The exceptional mass sensitivity of micro and nanomechanical resonators makes them interesting for applications such as for the detection of airborne nanoparticles. The use of nanoparticles in commercial applications has increased and personal monitoring devices for the assessment of nanoparticle exposure doses are demanded due to the still unknown toxic effects [6]. Recently, nanobeam based sensors have been used as mass spectrometers detecting single bio molecules [7]. By measuring resonant frequency shifts of the first resonant mode caused by the impact of individual molecules Naik *et al*. could calculate the mass of the molecules by building histograms of event probability versus frequency-shift amplitude. This technique demands a complex post-measurement histogram-based analysis which hinders its implementation in a real-time and portable nanoparticles monitor.

In this work, we present a method which allows the sensing of the position and mass of individual particles every time a mass adsorption event occurs. With this technique, the mass of single particles can be determined in air and vacuum in real-time without the need of a post-measurement analysis, enabling mass spectrometry of particles with distributed masses. By measuring the resonant frequency shifts of the first two bending modes, the position and mass of a particle attached to a micro cantilever can be calculated, as we reported in an earlier work [8]. With resonant cantilevers, the position and mass calculation requires the relatively complex and time consuming computation of the minima of a specific functional which complicates an implementation in a easy to use real-time mass spectrometer. This problem can be solved by using strings rather than cantilevers. Strings are mechanically more stable, which in particular results in a higher fabrication yield compared to cantilevers, and intrinsic energy loss mechanisms are very small [9]. That is, they can have quality factors of over a million in vacuum [10]. The main reason for choosing strings is their simple bending mode shape function compared to cantilever beams. This allows the derivation of a closed form solution for the particle mass and position.

## 2. Theory

**Figure 1.**
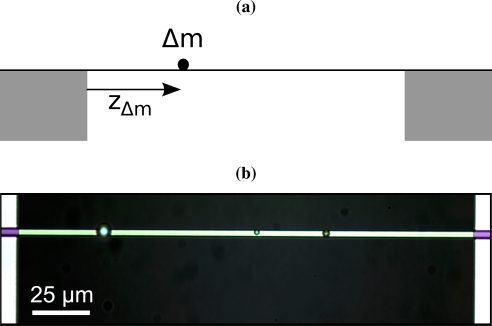
(a) Schematic side view of a string with a single particle of mass Δ*m* positioned at *z_Δm_*. (b) Microscope top view of a silicon nitride string with 3 different particles attached. From left to right: 6 *μm* Polybead, 2 *μm* Polybead, and 2.8 *μm* Dynabead.

For a string with length *L*, the mode shape function is given by

(1) $$U_{n}(z)=\text{sin}\left(\frac{n\pi z}{L}\right)$$

where *n* is the mode number. Figure 1a shows the examined scenario where a string with mass *m*_0_ is loaded by a point mass Δ*m* positioned at *z*_Δ*m*_. At resonance, the time average kinetic energy of the string and the loaded mass equal the time average strain energy of the string. The kinetic energy of a string is given by

(2) $$E_{\mathrm{kin}}=\int_{V}\frac{1}{2}{\rho \omega }_{n,\Delta m}^{2}a_{n}^{2}U_{n}^{2}(z)\mathrm{dV}$$

where *ρ* is the mass density, *ω_n,Δm_* is the resonant frequency of the loaded string and *a_n_* is the modal amplitude at mode *n*. With $\int_{0}^{L}{\text{sin}}^{2}\left(\frac{n\pi z}{L}\right)\mathrm{dz}=\frac{L}{2}$, Equation (2) becomes

(3) $$E_{\mathrm{kin}}=\frac{1}{4}m_{0}\omega_{n,\Delta m}^{2}a_{n}^{2}$$

The kinetic energy of the added point mass Δ*m* at position *z*_*Δm*_ is

(4) $$E_{\mathrm{kin},\Delta m}=\frac{1}{2}\Delta m\omega_{n,\Delta m}^{2}a_{n}^{2}U_{n}^{2}(z_{\Delta m})$$

Assuming that an added point mass does not alter the mode shape of the micro string, the strain energy does not change with the point mass adsorption. The strain energy is thus equal to the kinetic energy of the unloaded string

(5) $$E_{\mathrm{strain}}=\frac{1}{4}m_{0}\omega_{n}^{2}a_{n}^{2}$$

Therewith, the resonant frequency of a string with an attached small single mass can be derived by equalizing the kinetic with the strain energy *E_strain_* = *E_kin_* + *E_kin,Δm_* and becomes

(6) $$\omega_{n,\Delta m}^{2}=\omega_{n}^{2}{\left(1+2\frac{\Delta m}{m_{0}}U_{n}^{2}(z_{\Delta m})\right)}^{-1}$$

The point mass and its position are the unknowns of a defined second order system of equations based on Equation(6) for the first two bending modes. For the first bending mode (*n* = 1), Equation(6) can be solved for the position

(7) $$z_{\Delta m}=\frac{L}{\pi }\;\text{arcsin}\left(\sqrt{\frac{1}{2}\frac{m_{0}}{\Delta m}\left({\left(\frac{\omega_{1}}{\omega_{1,\Delta m}}\right)}^{2}-1\right)}\right)$$

The absolute string displacement is symmetrical and it does not make a difference on which half side a point mass is added to the string. The resulting frequency shift is the same. Therefore, the positions resulting from Equation(7) have only values from 0 to *L*/2.

The mass ratio of the point mass versus string mass can now be obtained by applying Equation(7) and Equation(1) in Equation(6) for the second mode (*n* = 2). By using the theorem $\left(\text{sin}(2\;\text{arcsin}\;x)=2x\sqrt{1-x^{2}}\right)$, a simple term for the mass ratio can be calculated.

(8) $$\frac{\Delta m}{m_{0}}=\frac{\frac{1}{2}{\left({\left(\frac{\omega_{1}}{\omega_{1,\Delta m}}\right)}^{2}-1\right)}^{2}}{\left({\left(\frac{\omega_{1}}{\omega_{1,\Delta m}}\right)}^{2}-1\right)-\frac{1}{4}\left({\left(\frac{\omega_{2}}{\omega_{2,\Delta m}}\right)}^{2}-1\right)}$$

The used theorem holds only if |*x*| < 1. It can easily be shown that this condition is fulfilled if *z_Δm_* < *L*/2 which is in agreement with the before mentioned symmetry of the string vibration.

Using Equation(7) and Equation(8), it is now possible to calculate the position *z_Δm_* and the mass Δ*m* of an individual point mass placed on an random spot on a string by measuring the resonant frequency shift of the first and the second bending mode for a string with a mass *m*_0_.

## 3. Experimental

For the experiments, silicon nitride strings are used. Since the particle mass calculation assumes a perfect sine mode shape, it is crucial to have micro strings which behave like a theoretical string as good as possible. Usually, strings fabricated by surface micromachining feature suspended anchor plates caused by the release etch. Such suspended anchors alter the mode shape of a string. Therefore, strings with a well defined clamping were fabricated by means of a bulk process. Silicon nitride is first deposited on a silicon wafer by LPCVD (low pressure chemical vapor deposition). The silicon nitride strings are then structured by a dry etch step. The front side with the structured strings is covered with a protective PECVD (plasma-enhanced chemical vapor deposition) silicon nitride layer. The strings are then released from the backside by means of a KOH (Potassium hydroxide) etch. In a last step, the PECVD silicon nitride cover layer is selectively removed in buffered HF (hydrofluoric acid) which is possible due to the large difference in the etch rate of PECVD and LPCVD silicon nitride. The strings obtained with this process feature a well defined clamping as can be seen in Figure 1b. The used strings are 216.4 *μm* long, 2.7 *μm* wide and 340 *nm* thick.

In an automated sensor, the first and the second bending mode would be simultaneously excited and the two resonant frequencies would be tracked real-time by frequency-locking. In this work for a proof of concept, single micro particles of three different weights were placed randomly on micro strings one after the other and the first two resonant frequencies were measured in between at any one time. For the measurements, 2 *μm* and 6 *μm* polystyrene beads (Polybeads) and 2.8 *μm* magnetic beads (Dynabeads) with masses of 4.8 ± 0.18 pg, 111.8 ± 14.4 pg, and 14.9 ± 5.4 pg, respectively, were used. The particles were picked and placed using an etched tungsten tip with a diameter of roughly 1 *μm* mounted to a high-precision xyz-stage. Micro particles placed on micro strings by this method can be seen in Figure 1b.

The resonant frequencies were detected optically at atmospheric pressure at room temperature with a laser-Doppler vibrometer (MSA-500 from Polytec GmbH) by measuring the thermal-noise resonance peaks. The laser spot size was in the order of the string width.

In order to verify that the double-clamped micro beams mechanically behave string-like, the frequencies of higher bending modes were measured. The resonant frequencies of Euler-Bernoulli beams whose resonant behavior is determined by the flexural stiffness are proportional to $\lambda_{n}^{2}$. For a double-clamped beam, the λ*_n_* values for *n* = 1, 2, 3, *n* > 3 are 4.7300, 7.8532, 10.9956, (2*n* + 1)π/2, respectively. In contrast, the resonant frequency of a string is defined by the tensile pre-stress and the resonant frequencies of higher bending modes are a multiple of the first mode [11]. The string nature of the double-clamped silicon nitride micro beams has been verified by testing the linear relation between the mode number and the corresponding resonant frequencies. The average first mode resonant frequency is 628.5 kHz and the frequency ratios of the second and third harmonic are 2.004 ± 0.002 and 3.027 ± 0.005, respectively. Thereby, a tensile stress of 222 MPa was evaluated.

## 4. Results & Discussion

**Figure 2.**
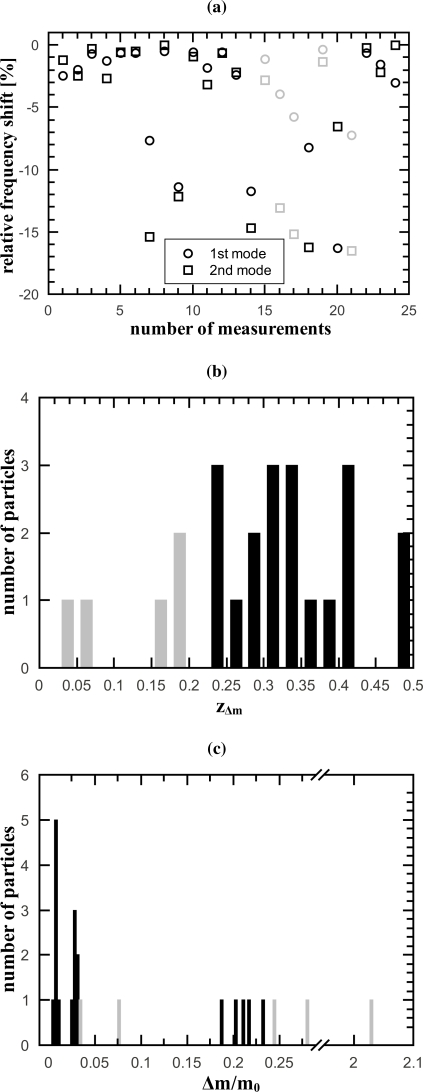
(a) Measured relative frequency shifts of the first and second bending mode. Histograms of number of particles (b) versus particle positions Equation(7) for *L* =1and (c) versus mass ratio Equation(8) for the three different types of micro particles.

**Figure 3.**
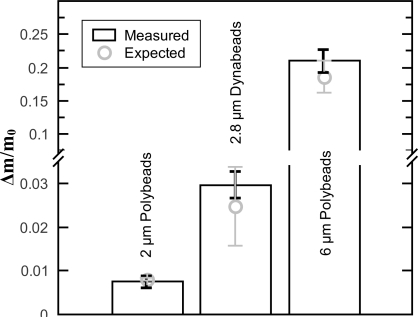
The average mass ratio Equation(8) for the three different types of micro particles compared to the expected values. For the string mass calculation a mass density for silicon nitride of *ρ* = 3, 000 kg/m^3^ was assumed. The black error bars represent the standard deviation of the different measurements. The gray error bars represent the error coming from the particle diameter deviation.

Figure 2 shows the measured relative frequency shifts and the histograms of the number of particles versus the calculated positions and mass ratios. For the calculations of the positions (Figure 2b) the beam length was set to *L* = 1. Since the bending mode shape of a string is symmetrical, as discussed in Section 2, the *z_Δm_* values range from 0 to 0.5. In the mass ratio histogram in Figure 2c, the three peaks for the three different particles types are clearly visible. Whereas the first two peaks for the smaller particles are very distinct, the peak for the heaviest particles is broader. This is mainly due to the constant small step size of the histogram. In Figure 3, the average mass ratios of the three different particle types are compared to the expected mass ratios.

For the proof of concept rather large micro beads were used. The 6 *μm* Polybeads cause relative frequency drops of up to 16 % as can be seen in Figure 2a. It has been shown experimentally that the assumption used for the derivation of (5) is valid for a mass ratio of Δ*m*/*m*_0_ = 0.0084 [8]. With the 6 *μm* Polybeads the expected mass ratio is Δ*m*/*m*_0_ = 0.186 which is significantly higher. When comparing the calculated resonant frequency from the model (6) to FEM simulations (made with Comsol 4) a maximal relative error of the resonant frequency of 1.2% is found for the 6 *μm* Polybeads. FEM simulations show that the error becomes smaller for smaller particles. The weight of the used 6 *μm* beads is at the limit where the model is becoming inaccurate. But the error from the model still is one order of magnitude smaller than the measured frequency shifts. The obtained average mass ratios in Figure 3 are precise and correspond to the expected values which shows that the presented model is valid even for the relatively heavy 6 *μm* Polybeads. The slight error in the mass ratio accuracy might be due to the uncertainty of the string mass.

For the calculation of the average mass ratio all the particles near the clamping have been discarded. By filtering all particles with *z_Δm_* < 0.2, all outliers in Figure 2c have been detected and the mass calculation becomes more precise. The discarded measurements are colored gray in Figure 2. The mass ratio Equation(8) becomes inaccurate if the denominator gets small and reaches the order of frequency measurement and model errors. It can be seen that the denominator in Equation(8) approaches zero if *z_Δm_* approaches zero. The frequency measurement error is constant (±0.03% from the number of FFT lines of the vibrometer) whereas the model error becomes larger for heavier particles, as has been shown with FEM simulations. The model error thus is dependent on the particle mass. Since the mass of the particles can be inaccurate before filtering it is therefore not possible to dynamically adjust the critical *z_Δm_* value for every measurement. In our work we chose a global value of 0.2 arising from the model error of 1.2% for the 6 *μm* beads. The critical *z_Δm_* value can be calculated by equalizing the denominator in Equation(8) with the error from the model.

(9) $${\left(\frac{1}{1-0.012}\right)}^{2}-1<2\frac{\Delta m}{m_{0}}\left({\text{sin}}^{2}(\pi z)-\frac{1}{4}{\text{sin}}^{2}(2\pi z)\right)$$

Thereby a critical *z_Δm_* value of around 0.16 is found for the 6 *μm* Polybeads. This value is close to the 0.2 used for our measurements.

The measured resonant frequencies of the higher harmonics accurately obey the string model (see Section 3) and it can be assumed that the mode shapes also accurately obey the sinusoidal mode shapes. If the string would behave beam like, the vibrational amplitude near the clamping would be small due to the clamping conditions and correspondingly the measured relative frequency shift would be small too, which is not the case as can be seen in Figure 2a. Therefore it can be concluded that the effect of discarding all particles located close to the clamping is based on the above discussed effect and not on mode shape imperfections near the clamping.

The average relative frequency shifts caused by the 2 *μm* Polybeads are 0.6% and 0.4% for the first and second bending mode, respectively. It has been shown that micro beam resonators can have a frequency resolution of around 4 × 10^−4^% at atmospheric pressure [12] and 6 × 10^−5^% [7] in high vacuum. Based on these frequency resolutions, it is expected that with the used micro strings masses of femtograms in air and hundreds of attograms in high vacuum can be detected. In order to increase the sensitivity for the detection of smaller particles it is necessary to decrease the string mass *m*_0_. A typical nano beam resonator has a mass as low 1 pg which increases the mass sensitivity by three orders of magnitude. This allows a mass resolution in the attogram range in air [12], which makes it possible to detect nanoparticles with a diameter of 10 nm with a typical mass of Δ*m* > 1 ag.

The computing time for the presented closed-form solution was compared to the minimization method presented in [8]. A Matlab script of both methods was executed on a personal computer (linux, 2.4 GHz processor, 2 GB memory). The computation of the mass ratio with the presented closed-form expression took 0.3 ms whereas the minimization algorithm took 3.5 s to finish. Thus, the presented method is 4 orders of magnitude faster, enabling the real-time detection of particles with an adsorption rate of more than 3,000 particles per second whereas the minimization method can only detect adsorption rates of a few particles per minute.

## 5. Conclusions

We have presented a simple analytical closed-form solution for the calculation of the position and the mass ratio of a point mass on a string. This method enables a quick and accurate serial detection of individual point masses adsorbing on a string resonator. The method has been successfully tested by placing micro particles with different known masses on silicon nitride micro strings. It has been shown, that the precision of the mass detection can be improved by neglecting all the particles that are placed near the clamping where the mass resolution is poor. The presented method enables real-time mass spectrometry based on micro- or nanomechanical resonant strings which can be used for applications such as the detection of airborne nanoparticles.
